# Quantum key distribution over 120 km using ultrahigh purity single-photon source and superconducting single-photon detectors

**DOI:** 10.1038/srep14383

**Published:** 2015-09-25

**Authors:** Kazuya Takemoto, Yoshihiro Nambu, Toshiyuki Miyazawa, Yoshiki Sakuma, Tsuyoshi Yamamoto, Shinichi Yorozu, Yasuhiko Arakawa

**Affiliations:** 1 Devices & Materials Laboratory, Fujitsu Laboratories Ltd., 10-1 Morinosato-Wakamiya, Atsugi, Kanagawa 243-0197, Japan; 2 Smart Energy Research Laboratories, NEC Corporation, 34 Miyukigaoka, Tsukuba, Ibaraki 305-8501, Japan; 3 Institute for Nano Quantum Information Electronics, The University of Tokyo, 4-6-1 Komaba, Meguro-ku, Tokyo 153-8904, Japan; 4 National Institute for Materials Science (NIMS), 1-1 Namiki, Tsukuba, Ibaraki 305-0044, Japan; 5 Institute of Industrial Science, The University of Tokyo, 4-6-1 Komaba, Meguro, Tokyo 153-8505, Japan

## Abstract

Advances in single-photon sources (SPSs) and single-photon detectors (SPDs) promise unique applications in the field of quantum information technology. In this paper, we report long-distance quantum key distribution (QKD) by using state-of-the-art devices: a quantum-dot SPS (QD SPS) emitting a photon in the telecom band of 1.5 μm and a superconducting nanowire SPD (SNSPD). At the distance of 100 km, we obtained the maximal secure key rate of 27.6 bps without using decoy states, which is at least threefold larger than the rate obtained in the previously reported 50-km-long QKD experiment. We also succeeded in transmitting secure keys at the rate of 0.307 bps over 120 km. This is the longest QKD distance yet reported by using known true SPSs. The ultralow multiphoton emissions of our SPS and ultralow dark count of the SNSPD contributed to this result. The experimental results demonstrate the potential applicability of QD SPSs to practical telecom QKD networks.

Over the past decade, advances in sources, operational devices, and detectors have attracted the attention of many researchers in the field of quantum information technology. In particular, single-photon sources (SPSs) and single-photon detectors (SPDs) are key devices for enabling practical applications; e.g., quantum key distributions (QKDs)^1^. Telecom-band SPSs and SPDs are of special interest because the existing telecom backbone networks exhibit a minimal transmission loss around 1.55 μm.

For a good while, attenuated lasers and avalanche photodiodes (APDs) have been used for practical telecom-band SPSs and SPDs^2^,^3^,^4^,^5^,^6^. Potential attacks exploiting the nonideality of SPSs were pointed out^7^,^8^, but were soon countered with decoy-state QKDs^9^,^10^,^11^. However, because legitimate users need to precisely control the average number, μ, of photons in each pulse, statistical effects arising from the finite key size and ambiguity of μ might be so severe that a significant shrinking of the sifted key might be required^12^. In addition, the complexity of the protocol and system may contain loopholes. For example, it has been pointed out that a source attack against decoy-state QKD is possible if Eve can exploit the source’s phase information^13^,^14^. In general, QKD based on a true SPS is desired because it relaxes relevant requirements and reduces the risk of loopholes resulting from a gap between actual and ideal implementations. Currently, transmission of secure keys over 50 km of in-lab fiber is the record of standard QKD based on a true SPS; that experiment used a telecom-band InAs/InP quantum dot (QD) SPS and conventional InGaAs avalanche SPD (ASPD)^15^. The transmission distance was limited by the residual multiphoton emission of the SPS and dark count of the SPD. Extending the transmission distance is of course a motivation for further studies. In this paper, we report secure key distribution up to 120 km (i.e., covering a large metropolitan area) by using a true SPS and the standard QKD protocol. To achieve this, a high-purity SPS was needed, i.e., one having a small probability of emitting more or fewer than 1 photon. This is a challenging problem, because the goals of enhancing single-photon efficiency and suppressing multiphoton emission are incompatible in many SPSs^16^,^17^,^18^,^19^,^20^,^21^.

In this work, we demonstrate secure key distribution up to 120 km by using an ultrahigh purity QD SPS and a superconducting nanowire SPD (SNSPD)^22^,^23^,^24^,^25^. The effective suppression of multiphoton emission with second-order correlation values down to *g*^(2)^(0) ~ 0.002 was realized by the combination of a quasi-resonant optical excitation to the p-shell state of a QD and an excitation-pulse-width compression technique. Because the single-photon pulses have finite pulse widths of ~1 ns, the low dark count and nongated mode operation of the SNSPD enabled to significantly enhance the signal-to-noise (S/N) ratio of our QKD system. Finally, we demonstrate that our true SPS has the potential to extend the transmission distance over 200 km by accounting for the additional improvement of *g*^(2)^(0) and source efficiencies.

## Results

### Time-bin encoding QKD system

**Figure 1:**
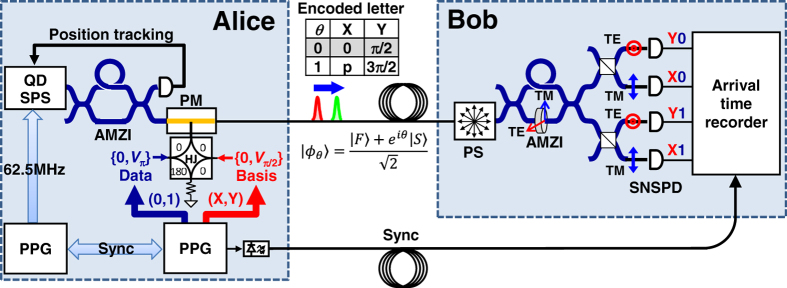
Experimental setup of single-photon QKD. Test-bed system, i.e., a time-bin encoding QKD system based on the standard BB84 protocol. PPG: pulse pattern generator, HJ: hybrid junction. The entire system was operated at the repletion rate of 62.5 MHz.

As a test-bed, we used a time-bin encoding QKD system^26^,^27^,^28^,^29^ based on the standard Bennett-Brassard 1984 (BB84) protocol^1^. The experimental setup is shown in Fig. 1. It is based on two identical asymmetric Mach-Zhender interferometers (AMZIs) fabricated with a planar light-wave circuit, for Alice and Bob^15^,^27^. The two AMZIs defined a qubit space for quantum coding. They were precisely temperature-controlled so that no phase information of source was necessary for establishing a relevant common reference frame during QKD operation (in contrast to ref. 13). At Alice’s site, an SPS emitted an optical pulse. The optical pulse passed through the first AMZI, which converted it into a traveling pair of double pulses with a 5-ns time interval and fixed polarization. One output of the AMZI was used for time-bin qubit, and the other was used for tracking the SPS’s position. The relative phase of the double pulse was subsequently modulated by using a phase modulator (PM) with a randomly chosen value from *θ* = {0, π/2, π, 3π/2}. After traveling through a fiber core in two-core single mode fiber (SMF), a double pulse arrived at Bob’s site, and its polarization was randomized by using a polarization scrambler (PS). Then, the double pulse was fed into the second AMZI. As a result of the waveguide’s polarization mode dispersion, the TM and TE modes of this AMZI worked as an analyzer for the X-basis associated with *θ* = {0, π} and Y-basis associated with *θ* = {π/2, 3π/2}. The PS and AMZI, followed by polarization beam splitters (PBSs) for distinguishing the TE and TM modes, constituted a BB84 decoder based on a passive basis choice. The arrival port of the photon yielded both the chosen basis and the measurement result, and arrival was detected by four SPDs connected to each port. The photon arrival port and time were recorded by using a time-interval analyzer (TIA).

### Ultrahigh purity QD SPS and low-noise SNSPD

**Figure 2:**
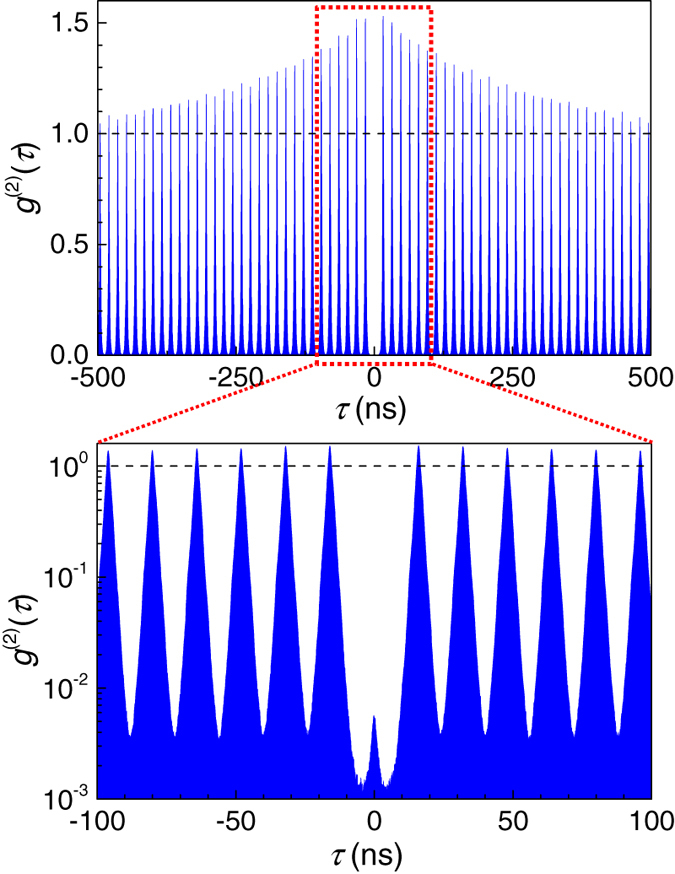
Photon correlation measurement. Results of correlation measurements of emitted photons associated with this experiment; *g*^(2)^(τ) is plotted as a function of the time delay τ of the arrival time of the photons. We chose *g*^(2)^(0) = 0.0051 and 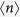 = 0.05 at the repetition rate of 62.5 MHz. The lower trace is a magnification of the central part of the upper trace around τ ~ 0, and *g*^(2)^(0) is plotted on a logarithmic scale.

We refined our SPS and SPD for our novel system. Our SPS is an optically excited self-assembled InAs/InP QD^30^ with an optical horn structure^31^,^32^. To ensure high photon emission efficiency and low multiphoton emission, the QD is excited by quasi-resonant optical pulses generated from a distributed feedback laser diode (DFB LD) with a tunable dispersion compensator (see Supplementary Fig. S1 online). The emitted photon with a wavelength of 1580.5 nm is typically characterized by two parameters, 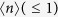 (the average number of photons in the emitted pulse coupled to the fiber) and *g*^(2)^(0) (the second-order correlation function at zero time delay). Both of these parameters depend on the SPS’s operating conditions. We created a compressed optical excitation pulse of about 10 ps (shorter than that used in the previous system) for suppressing multiphoton emissions owing to multiexciton excitations. We also increased the repetition rate of the SPS from 20 to 62.5 MHz, by taking account of the previously established practical system working on a 62.5 MHz clock^33^. The best result for the correlation measurements of emitted photons by using the Hanbury-Brown-Twiss setup is reported in Supplementary Fig. S2 online, where we obtained *g*^(2)^(0) = 0.002 (after careful background subtraction) and 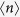 = 0.03. In the current demonstration, we relaxed the conditions by widening the band pass filter’s bandwidth immediately after the SPS from 0.3 to 0.7 nm. As shown in Fig. 2, we obtained *g*^(2)^(0) = 0.0051 and 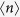 = 0.05 at the repetition rate of 62.5 MHz. Although this condition is not optimal for multiphoton suppression, the value of *g*^(2)^(0) is almost one order of magnitude smaller than the value in the previous experiment, while 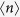 is only slightly reduced (0.06 → 0.05)^14^.

We replaced ASPD with a four-channel SNSPD (SCONTEL, FCOPRS-00-15). The SNSPD is more advantageous than the ASPD, because it has both high detection efficiency and low dark count. Its performance depends on the operating conditions, in particular the operation temperature and the applied bias voltage. Here, we chose conditions that would simultaneously ensure a dark count rate under 20 cps and quantum efficiency of 10%. One of the important differences between the ASPD and SNSPD is that the former operates in a gated mode, whereas the latter operates in a nongated mode. As will be discussed later, this feature and the small dark count of the SNSPD helped to significantly improve the S/N ratio of our QKD system.

### Demonstration of single-photon QKD

**Figure 3:**
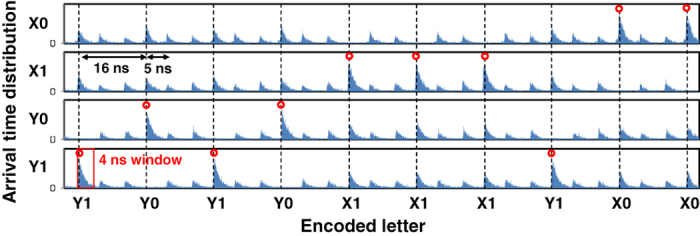
Histograms of detected signal. Histograms showing the arrival time distributions of photons for the SNSPDs. Only ten bits of data are shown here. Encoded letters are shown in temporal order on the abscissa axis. The predefined temporal positions associated with meaningful events are shown as vertical dashed lines. Successfully decoded events are shown as circles.

For the demonstration, four-letter codes randomly chosen from {X0, X1, Y0, Y1} and cyclic with a 100-bit period (1.6 μs) were each encoded into a series of photon pulses. The time reference between Alice and Bob was established by sending synchronizing laser pulses in parallel through a two-core SMF. The histograms in Fig. 3 show the arrival time distributions of the photon for the four SNSPDs, where only parts of data are shown. The distributions are localized around the predefined temporal positions with finite widths. This temporal width mainly reflects the finite lifetime (~1 ns) of the photon emission in our QD SPS. We observed that the width was independent of the transmission distance. This indicates that the photon pulse was chirp-less, i.e., our system was completely dispersion-free. Raw keys were generated by postselecting the useful events from such distributed events. This postselection clearly depends on the window size of the event selection. The larger the window size is, the more we can select the events and increase the raw key rate as well as the error rate. This is because, in our time-bin optics, there are satellite events before and after the useful events, which yield 50% of the errors. Therefore, enlarging the window too much may decrease the secure key rate. For this reason, we chose a window size of 4 ns. The resulting quantum efficiency η_eff_ and dark count probability *d*_B_ per window were slightly smaller than 10% and 1 × 10^−7^, respectively. Moreover, the resulting effective S/N ratio of our SNSPD was about 60.8 dB. By contrast, the ASPD window is usually very small, e.g. around 1 ns, because it is usually operated in a gated mode to keep the dark count at a reasonable level. Moreover, we could only apply triangular gate pulses (rather than rectangular ones) to the ASPD for such a short temporal range because of the limited bandwidth of the peripheral circuit. This, together with the finite widths of the photon arrival time distributions, severely limited the quantum efficiency per gate of the ASPD. In our case, quite a small fraction of arriving photons could be postselected as useful events. The resulting effective S/N ratio was below 50 dB, and this is why we replaced the ASPD with an SNSPD that improved the S/N ratio of the SPD by more than one order of magnitude.

**Figure 4:**
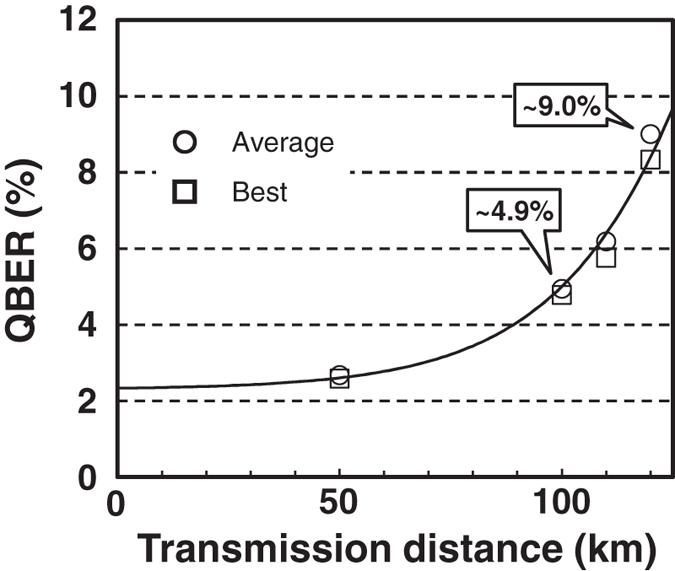
QBER. Measured QBER as a function of transmission distance. Data sent through 50, 100, 110, and 120 km-long SMF spools are shown. The rectangles and circles are, respectively, the best data and the average of the top ten data during the experiment. The solid line is the fit for obtaining some of the experimental parameters.

**Figure 5:**
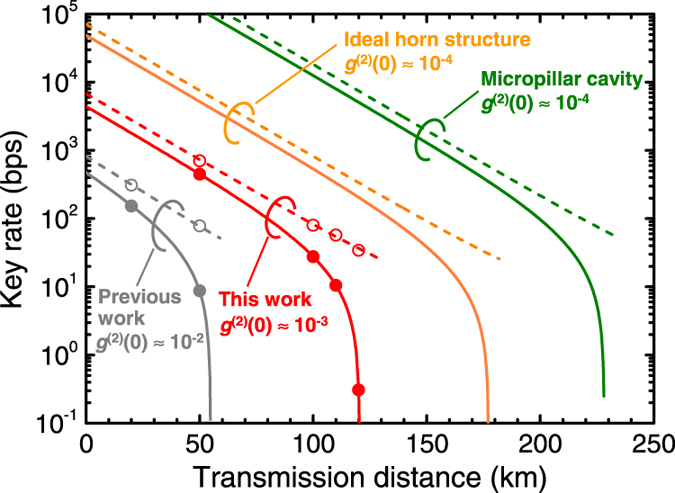
Key rate as a function of the transmission distance. Measured raw key rates plotted as a function of transmission distance (red open circles and red dotted line) together with the estimated secure key rates (red closed circles and red solid line). The previous result^15^ (50-km-long transmission) is shown for comparison. Orange dotted (solid) and green dotted (solid) lines shows raw (secure) key rates for ideal horn structure and micropillar cavity structure, respectively (see text).

To summarize, we reduced the multiphoton emission of our SPS by more than one order of magnitude and increased the S/N ratio of our SPD by more than one order of magnitude relative to the previous system. The latter improvement significantly contributed to reducing the quantum bit error rate (QBER). Figure 4 shows the measured QBER after transmission through 50, 100, 110, and 120 km-long two-core SMF spools. In all the distances, the QBER was below 10%, which suggests the possibility of secure key distribution up to 120 km. To prove this prediction, we need to analyze the secure key rate while accounting for the finite value of evaluated *g*^(2)^(0). We performed security analysis according to the GLLP theory^34^,^35^. To this end, we needed to determine several system parameters. Some parameters were measured directly by using classical light, while others were obtained by fitting the measured QBER (Fig. 4). The relevant parameters are listed in Table 1. The error correction algorithm was assumed to consume 1.2 times the number of bits attainable in the Shannon limit. The red dotted and solid lines in Fig. 5 show the results for the raw and estimated secure key rates for a series of experiments, respectively. The estimated secure (raw) key rate was about 27.6 bps (79.9 bps) and 0.307 bps (34.3 bps) at 100 km and 120 km, respectively. It should be noted that the theory suggests that we could further extend the maximal range of secure keys distribution by ~10 km under the same *g*^(2)^(0) if we could increase 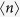 from 0.05 to ~0.10, because the leading factor limiting the maximal range in this demonstration is the detector’s noise (see Supplementary Fig. S3 online). This result clearly indicates that our novel system has the potential to distribute secure keys over 120 km, corresponding to the size of a typical metropolitan area.

## Discussion and Outlook

From a practical viewpoint, it is important to discuss the potential performance of the time-bin encoding QKD system by accounting for further improvement of QD SPS and SNSPD. The orange dotted (solid) and green dotted (solid) lines in Fig. 5 show the calculated raw (secure) key rate based on the GLLP theory for an ideal horn structure^30^ and a pillar microcavity structure^36^, respectively. In both cases, the *g*^(2)^(0) is assumed to be ~10^−4^, which is lower than the current QD SPS by one order of magnitude. This might be attainable by further shortening the excitation pulses (see Supplementary Information). As for the detector’s performance, we assumed a dark count rate of 18 Hz at a system detection efficiency of 40%, which is within the current experimental reach^37^. For the ideal horn structure (i.e., orange dotted and solid lines in Fig. 5), we assumed 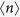 = 0.175 taking into account the maximal photon extraction efficiency of 35% and 3-dB loss between the lens and a SMF core. For the repetition rate of 62.5 MHz, which is the same as in the above QKD demonstration, the secure key rate was estimated as ~40 bps at 150 km. The distance is comparable to the record of phase-encoding QKD system using decoy states^38^. In this case, the radiative lifetime of ~1 ns for the exciton state limits the maximal repetition rate to several hundred megahertz. Recently, we have proposed a novel hybrid pillar microcavity structure in which a large Purcell factor (up to 110) with output efficiency of ~60% can be obtained^36^. If such an SPS is integrated into our system, reduced timing jitter of the single-photon emission can contribute to extending the maximal repetition rate into gigahertz range. In addition, it enables to narrow the temporal postselection window of SNSPDs, thus reducing the influence of background noise. The green solid line in Fig. 5 shows the calculated secure key rate at the repetition rate of 1 GHz, assuming that the timing jitter of emitted photons is reduced by 1/10 while keeping *g*^(2)^(0) at 10^−4^ and 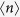 at 0.175. The simulation indicates that the predicted secure bit rate reaches ~100 bps at 200 km, which is similar to the recent polarization-encoding QKD system using decoy states^39^. Note that the condition of the source performance (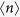 = 0.175 and *g*^(2)^(0) = 10^−4^) assumed for these simulations is greatly mitigated compared with ideal SPS (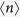 = 1 and *g*^(2)^(0) = 0). This shows that the possibility of pursuing *g*^(2)^(0)-engineering to obtain high-purity QD SPS offers unconditionally secure QKD based on single-photon technology as well as high throughput comparable to the current QKD system based on coherent light, even for SPSs with moderate efficiencies.

In conclusion, we demonstrated secure QKD over 100 km by using the true SPS. This achievement was by virtue of the improved SPS and SPD. By applying a very short and resonant excitation pulse to optically excite our InAs/InP QD, we obtained pulsed photons emitting at 1.58 μm with ultralow *g*^(2)^(0) and relatively high 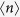. Our QKD system also used SNSPDs, which greatly improved the S/N ratio. The raw and secure key rates at 100 km were about 79.9 and 27.6 bps, respectively. The latter value was more than threefold larger than the rate obtained in the previous 50-km-long QKD experiment^14^. Furthermore, the maximal range of secure QKD reached 120 km, for which we achieved the raw and secure key rates of 34.3 bps and 0.307 bps, respectively. This result demonstrates that our QD-based 1.5-μm SPS could be used in future telecom QKD networks.

## Additional Information

**How to cite this article**: Takemoto, K. *et al.* Quantum key distribution over 120 km using ultrahigh purity single-photon source and superconducting single-photon detectors. *Sci. Rep.* **5**, 14383; doi: 10.1038/srep14383 (2015).

## Supplementary Material

Supplementary Information

## Figures and Tables

**Table 1:** Relevant parameters necessary for the security analysis according to the GLLP theory.

Relevant parameters	Symbol	Value
SPS parameters	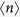	0.05
	*g*^(2)^(0)	0.0051
SPD parameters	*η*_eff_	0.096
	*d*_B_	4 × 0.75 × 10^−7^
QBER due to optical misalignment	QBER_opt_	2.3%
Optical loss of Alice’s system	*η*_A_	7.43 dB
Optical loss of Bob’s system	*η*_B_	3.00 dB
Optical loss of fiber	*α*	0.194 dB/km
