# Supplementary material for: Quantum key distribution over 120 km using ultrahigh purity single-photon source and superconducting single-photon detectors
Source: Sci Rep. 2015 Sep 25;5:14383. doi: 10.1038/srep14383 (PMC4585911; doi:10.1038/srep14383)
Supplement: Supplementary Information [file srep14383-s1.pdf]

Supplemental Information for
Quantum key distribution over 120 km using ultrahigh purity single-photon source and superconducting single-photon detectors

KAZUYA TAKEMOTO[1], YOSHIHIRO NAMBU[2], TOSHIYUKI MIYAZAWA[3], YOSHIKI SAKUMA[4], TSUYOSHI YAMAMOTO[1], SHINICHI YOROZU[2], AND YASUHIKO ARAKAWA[3,5]

[1]Fujitsu Laboratories Ltd., 10-1 Morinosato-Wakamiya, Atsugi, Kanagawa 243-0197, Japan
[2]Nano Electronics Research Laboratories, NEC Corporation, 34 Miyukigaoka, Tsukuba, Ibaraki 305-8501, Japan
[3]Institute for Nano Quantum Information Electronics, The University of Tokyo, 4-6-1 Komaba, Meguro-ku, Tokyo 153-8904, Japan
[4]National Institute for Materials Science (NIMS), 1-1 Namiki, Tsukuba, Ibaraki 305-0044, Japan
[5]Institute of Industrial Science, The University of Tokyo, 4-6-1 Komaba, Meguro, Tokyo 153-8505, Japan

1. Generation and characterization of single photons

The experimental setup for the photon autocorrelation measurement is shown in Fig. S1. The QD was optically excited by using a gain-switched distributed feedback laser diode (DFB LD). To characterize the QD SPS, the DFB-LD was driven at the repetition frequency of 20–100 MHz by using a pulse-pattern generator (PPG), as shown in Fig. S1(a). The driving electric pulses from the PPG were fed into the LD via a bias tee (not shown). The amplitude of the electric pulses ranged from 3.174 V to 3.500 V, depending on the repetition frequency. The LD wavelength was finely tuned by using a thermo-electric controller (TEC). Because the wavelength-tuning range of the LD was at most 1–2 nm, the coarse wavelength selection was performed by choosing an appropriate LD module (FITEL

FOL15DDBA-A81-19620-E) whose wavelength approximately matched the e_1h_1 state of the confined excitons in the single QD. After the TDC, a tunable saturable absorber (BATOP FC-SANOS-1530-TEC) suppressed unwanted temporal noise in the excitation light. The irradiated light was linearly polarized in the [110] direction of the QD sample by using the first fiber polarization controller (FPC1). The single-photon pulses (SPPs) generated at a 1580.5-nm wavelength were collected by a lens module, which included an objective lens with a numerical aperture of 0.55 (Olympus LM-Plan 50x), and were separated by using a wavelength-division multiplexer (WDM) coupler. To realize the spectral selection, we exclusively used two types of optical band-pass filters (BPFs) with different passband widths: one was a narrow-band filter with a high insertion loss (0.35-nm full width at half maximum (FWHM) with a 2.46-dB loss penalty), and the other was a wide-band filter with a low insertion loss (0.7-nm FWHM with a 0.23-dB loss penalty). Finally, linearly polarized SPPs were obtained by passing the single photons through the second fiber polarization controller (FPC2).

The electric energy structure of the QD was clarified in advance by using micro-photoluminescence (μ-PL) and micro-photoluminescence-excitation (μ-PLE) measurements (Fig. S1(b)). We observed well-isolated spectral peaks labeled by e_nh_m, which were attributed to the n-th excited electron and the m-th excited hole state[1]. For the quasi-resonant excitation, we chose the e_1h_1 (p-shell) state, which has the corresponding wavelength of 1527.5 nm. The inset of Fig. S1(b) shows the single-photon wave packet with a radiative lifetime of 1.12 ns.

To control the excitation laser's pulse width, we introduced a fiber compression system based on a tunable fiber Bragg grating (FBG). Because the gain-switched pulses had a relatively broad temporal width of 20–30 ps accompanied by negative frequency chirping, they could be temporally compressed by applying an appropriate normal dispersion[2]. By changing the dispersion with the tunable FBG, we continuously tuned the excitation pulse width. Figure S1(c) shows the typical temporal waveform for the input and output pulses measured by using an autocorrelator. Figure S1(d) shows the actual pulse widths, which were determined from the hyperbolic-secant ($Sech^2$) fit to the data, with respect to the dispersion parameter D. The minimal pulse width of 10.0 ps could be obtained with a -63-ps/nm dispersion, which was about 40% of the input pulse width. On the other hand, the spectral bandwidth was ~0.3 nm, which negligibly changed after pulse compression. The calculated time bandwidth product (TBP) at D = -63 ps/nm was 0.37, close to the transform-limited $Sech^2$ pulse value of 0.315.

To characterize $g^{(2)}(0)$, we constructed a Hanbury Brown and Twiss (HBT) photon correlation setup [3] by using a pair of superconducting nanowire single-photon detectors (SNSPDs) [4]. The bias current of the SNSPDs was precisely controlled so that each channel had detection efficiency of 18.0% at a dark count rate of ~20 cps. A 400-m-long optical delay line (corresponding to 2 μs) was inserted before one of the SNSPDs to the time origin in the autocorrelation histograms.

2. Excitation pulse width dependence of $g^{(2)}(0)$

Figure S2(a) shows the results of the photon autocorrelation measurement for four different

excitation pulse widths. The four histograms were accumulated for 16 hours. This relatively long accumulation time was needed for obtaining satisfactory signal-to-noise ratio. Note that the vertical axis in each graph is expressed by using the logarithmic scale. In all cases, strong anti-bunching behavior was observed at zero time delay even for a high excitation power corresponding to 95% of the QD saturation. The residual peaks at $\tau = 0$ could be clearly distinguished from the noise background, which was primarily owing to the multiphoton contribution. The peak heights increased with increasing excitation pulse width (Δt_p). To evaluate the true $g^{(2)}(0)$ (*i.e.* $g^{(2)}(0)$ reflecting only the multiphoton probability), we performed a detailed quantitative analysis of the obtained data. The calculated true $g^{(2)}(0)$ values were 0.0023, 0.0064, 0.0138, and 0.0173 for Δt_p = 10, 25, 60, and 80 ps, respectively, as shown in Fig. S2(b). The minimal $g^{(2)}(0)$ represents a 400-fold reduction in the probability of having more than one photon compared with a Poisson-distributed light. From Fig. S2(c), it can be deduced that $g^{(2)}(0)$ almost linearly depends on the excitation-pulse width. Note that the raw $g^{(2)}(0)$ is almost the same as the true $g^{(2)}(0)$. This similarity reflects the low-noise characteristics of the HBT measurement by using our SSPDs. The linear fit converges to zero in the $\Delta t_\mathrm{p} \to 0$ limit, eliminating the possibility that the finite value of $g^{(2)}(0)$ is primarily ascribed to a parasitic background such as unwanted photon emission from neighboring QDs. According to the theoretical model by Brouri *et al.* [5], the probability that the system is re-excited during each excitation pulse and that the succeeding second photon emission occurs during a single excitation cycle depends linearly on the

excitation-pulse duration for Δt_p ranging from 10 ps to 100 ps. Consequently, the finite $g^{(2)}(0)$ value predominantly stems from the re-excitation process, which could be technically suppressed by further compression of the excitation pulses. For example, the actual temporal width for the most-compressed excitation pulses (Δt_p = 10 ps) still deviates from the ideal value for Fourier-limited Sech^2-shaped pulses (Δt_p = 8.4 ps). This difference may arise from the presence of nonlinear chirp components. Therefore, higher-order fiber dispersion compensation[6] could further reduce the excitation-pulse width, resulting in improved multiphoton suppression. Alternatively, mode-locked lasers can be used for obtaining femtosecond to picosecond excitation pulses in the 1.5 μm band. From the extrapolation of the linear fit in Fig. S2(c), we can predict that ultrashort excitation pulses with ~500 fs duration enable to realize $g^{(2)}(0)$ of 10^{-4}.

3. Estimation of maximal range as a function of <n>

The improvements in the SPS and SPD were both important in this demonstration. To see this, we calculated the maximal range of secure QKD against $\langle n \rangle$. In Fig. S3, the dashed and dash-dotted curves are the maximal ranges that would be obtained if the SPSs were ideal ($g^{(2)}(0) = 0$) and if the SPDs were ideal SPD ($d_\mathrm{B} = 0$); the upper and lower plots are associated with the current and previous experimental conditions, respectively. The maximal range for non-ideal SPS and SPD (solid lines) is limited by the ideal SPS and SPD curves. The improved SPD reduces QBER and pushes the lower dashed line for the ideal SPS toward the upper one. By contrast, the improved SPS and improved SPD

are both required to push the lower dash-dotted line for the ideal SPD toward the upper one. This is because multi-photon emission and loss in the link opens a backdoor for Eve to attack[8,9]. Therefore, the current results are the product of both improvements. The maximal range for the current experimental conditions was about 120.4 km, *i.e.*, slightly longer than the transmission distance in the present demonstration. It was more than 118.9 km, even if we account for the background noise in the measured $g^{(2)}(0)$ and assume $g^{(2)}(0) = 0.0071$ as the worst case. The calculation also suggests that we can further extend the QKD range if we can increase $\langle n \rangle$ of our SPS. This is because a leading factor limiting the maximal range in this demonstration was the detector's noise.

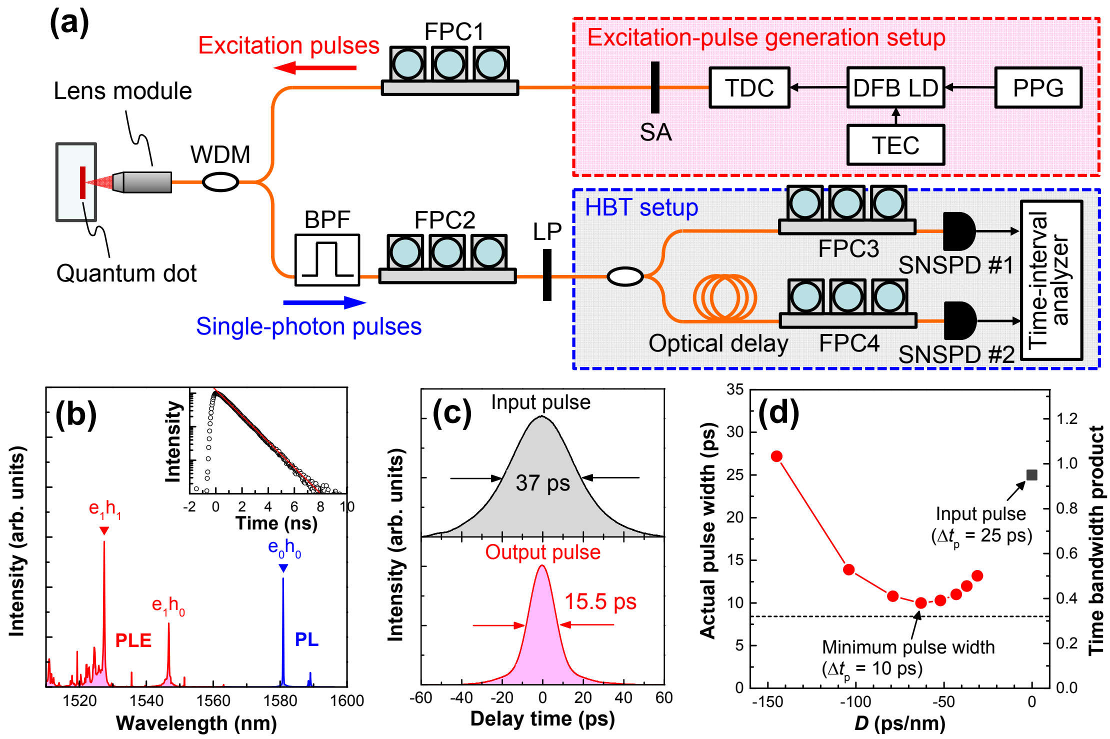

Figure S1:

Setup for single photon generation and characterization | (a) Schematic setup of the pulse compression and HBT measurement. (b) Micro PL and PLE spectra of a single InAs/InP QD. The inset shows the time-resolved waveform for the SPPs. (c) Time traces of the autocorrelation signals at zero bias current for *D* = 0 (input pulse) and -63 ps/nm. (d) Actual obtained pulse width and time bandwidth product as a function of *D*.

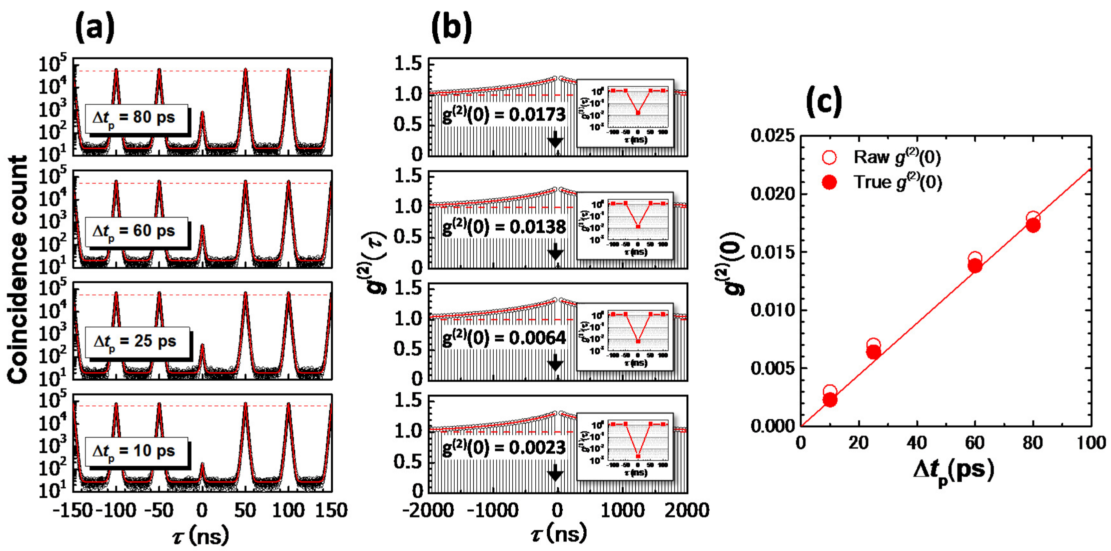

Figure S2:

Excitation-pulse-width dependence of $g^{(2)}(0)$ | (a) Autocorrelation histograms for four different excitation pulse widths at the repetition frequency of 20 MHz. Red solid lines show the least-squares fit to the data. The vertical axis in each graph is expressed on the logarithmic scale. (b) Normalized peak areas for the corresponding correlation histograms in (a) (in the linear vertical scale). The insets are the magnified linear-log plots of the ±110 ns temporal region. (c) Plot of the obtained $g^{(2)}(0)$ values against the excitation pulse width. The closed (open) circles denote the background subtracted (raw) data. The solid line shows the least linear fitting to the true (*i.e.* background subtracted) $g^{(2)}(0)$.

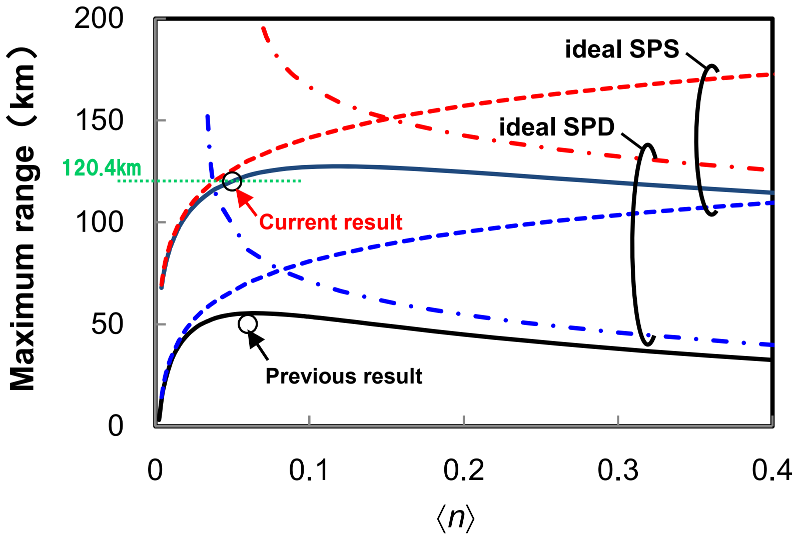

Figure S3:

Dependence of the maximal range on <n> | Calculated maximal ranges of secure QKD plotted (solid lines) as a function of the average number of photons <n> in the emitted pulse coupled to the fiber. The dashed and dash-dotted lines are those for the ideal SPS and SPD for the current (upper ones) and previous (lower ones) experimental conditions, respectively. Associated experimental data are shown as circles.

References

1. Miyazawa, T., Takemoto, K., Nakaoka, T., Saito, T., Hirose, S., Yokoyama, N. & Arakawa, Y. Effect of electronic structure on single-photon emission in InAs/InP quantum dot with quasi-resonant excitation. *Phys. Stat. Sol.* (c) **8**, 417-419 (2011).

2. Ahmed, K. A., Liu, H. F., Onodera, N., Lee, P., Tucker, R. S. & Ogawa, Y. Nearly transform-limited pulse (3.6 ps) generation from gain-switched 1.55 μm distributed feedback laser by using fibre compression technique. *Electron. Lett.* **29**, 54-56 (1993).

3. Hanbury-Brown, R. & Twiss, R. Q. Correlation between photons in two coherent beams of light. *Nature* **177**, 27-29 (1956).

4. Miki, S., Yamashita, T., Fujiwara, M., Sasaki, M. & Wang, Z. Multichannel SNSPD system with high detection efficiency at telecommunication wavelength. *Opt. Lett.* **35**, 2133-2135 (2010).

5. Brouri, R., Beveratos, A., Poizat, J.-P. & Grangier, P. Single-photon generation by pulsed excitation of a single dipole. *Phys. Rev. A* **62**, 063817 (2000).

6. Williams J. A. R., Bennion I. & Doran J. J. The design of in-fiber Bragg grating systems for cubic and quadratic dispersion compensation. *Opt. Commun.* **116**, 62-66 (1995).

7. Santori, C., Fattal, D., Vučković, J., Solomon, G. S., Waks, E. & Yamamoto, Y. Submicrosecond correlations in photoluminescence from InAs quantum dots. *Phys. Rev. B* **69**, 205324 (2004).

8. Brassard, G., Lütkenhaus, N., Mor, T. & Sanders, B. C. Limitations on practical quantum cryptography. *Phys. Rev. Lett.* **85**, 1330 (2000).

9. Lütkenhaus, N. Security against individual attacks for realistic quantum key distribution. *Phys. Rev. A* **61**, 052304 (2000).
